# Disseminated histoplasmosis in an immunocompetent patient - utility of skin scrape cytology in diagnosis: a case report

**DOI:** 10.1186/s13256-017-1543-5

**Published:** 2018-01-12

**Authors:** Tummidi Santosh, Kanchan Kothari, Shruti S. Singhal, Vyoma V. Shah, Roshni Patil

**Affiliations:** Department of Pathology, Seth Gordhandas Sunderdas Medical College and King Edward Memorial Hospital, Parel, Mumbai, Maharashtra 400012 India

**Keywords:** Histoplasmosis, Dimorphic fungus, Scrape cytology, Bird droppings, Disseminated

## Abstract

**Background:**

*Histoplasma capsulatum* is a dimorphic fungus predominately found in soils enriched with bird and bat excreta. Although several cases of histoplasmosis have been reported in India, diagnosis using cytology has been done in very few cases.

**Case presentation:**

We report here a case of disseminated histoplasmosis in a 46-year-old Indian man.

**Conclusion:**

Skin scrape cytology is a simple, safe, and rapid technique to establish the initial diagnosis, thus promoting early treatment and favorable outcome, in cutaneous fungal infections.

## Background

Histoplasmosis is an opportunistic fungal infection caused by inhalation of the microconidia (the mold form) of Histoplasma. They are found in soil and bird droppings. *H. capsulatum* is found in the Americas and the tropics and *Histoplasma dubosii* is prevalent in Africa [1]. In India, endemic cases of histoplasmosis have been reported in West Bengal (eastern India) and sporadic cases have been reported in southern India. Reports of histoplasmosis in nonendemic regions are very rare [2, 3]. Due to the varied and nonspecific clinical manifestations of histoplasmosis, most of the infections are misdiagnosed or underreported [4].

Skin scrape cytology is a simple, rapid and easy procedure which can be used as a diagnostic test for histoplasmosis. We report a rare case of disseminated histoplasmosis in an immune-competent patient.

## Case presentation

**Fig. 1:**
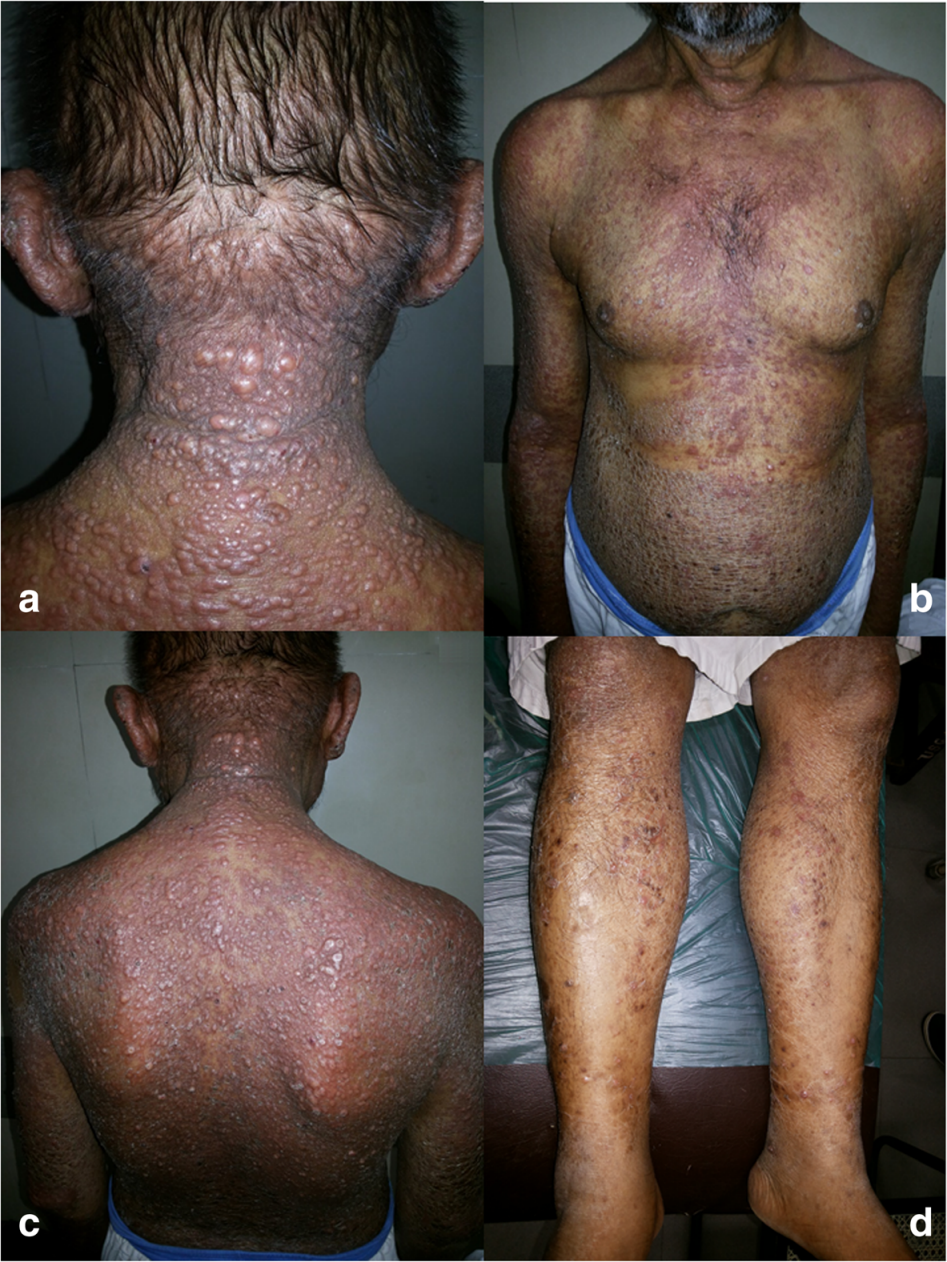
**a**, **b**, **c**, **d** Patient with multiple skin-colored to erythematous papules of varying sizes over face, nape of neck, oral cavity, scalp, upper limbs, abdomen, back, and lower limbs

A 46-year-old Indian man came to our hospital with complaints of fever and weight loss over the previous 5 months, multiple reddish papules for 2 months and dry cough for 1 month. By profession he used to sell tea underneath a tree and thus had been chronically exposed to bird droppings for 15 years. He had developed fever which was sudden in onset and high grade for which he was treated by a local physician as a case of malaria and was put on chloroquine. A month later, he developed multiple skin-colored to erythematous papules of size 0.5–1 cm over the nape of his neck followed by his face, oral cavity, scalp, upper limbs, abdomen, back and lower limbs (Fig. 1a, b, c, d). His fever was persistent. On examination, these papules were coalescing to form plaques and were associated with thickening of skin. Pus discharge and crusting was also seen. Our patient also had hoarseness of voice, nasal stuffiness, difficulty in breathing and deglutition. An abdominal examination revealed mild hepatomegaly. There was bilateral subcentimetric inguinal lymph node swelling but no cervical/axillary lymphadenopathy was seen. A chest X-ray was done which revealed no significant abnormality. Viral markers [human immunodeficiency virus (HIV)/hepatits C/hepatitis B] were negative. His hemoglobin level was 10.5 mg/dL, total leukocyte count was 15,500 mm^3^, platelet count was 400,000/uL and  peripheral smear showed normochromic normocytic anemia with reactive lymphocytosis. Erythrocyte sedimentation rate (ESR) level was 32 mm/1^st^ hour. Random sugar level was 99 mg%; liver function test: serum bilirubin 0.7 mg/dL(T), 0.4 mg/dL(D); SGOT 90Iu/L; SGPT 39Iu/L; serum alkaline phosphatase (ALP) 197 Iu/L; C-reactive protein (CRP) 150 mg/dL; antinuclear antibody (ANA) negative; serum urea 15 mg/dL; serum creatinine 0.9 mg/dL; serum Na 135 mmol/L; serum K 4 mmol/L. He had no history of smoking or alcoholism. There was no other significant medical or surgical history.

**Fig. 2:**
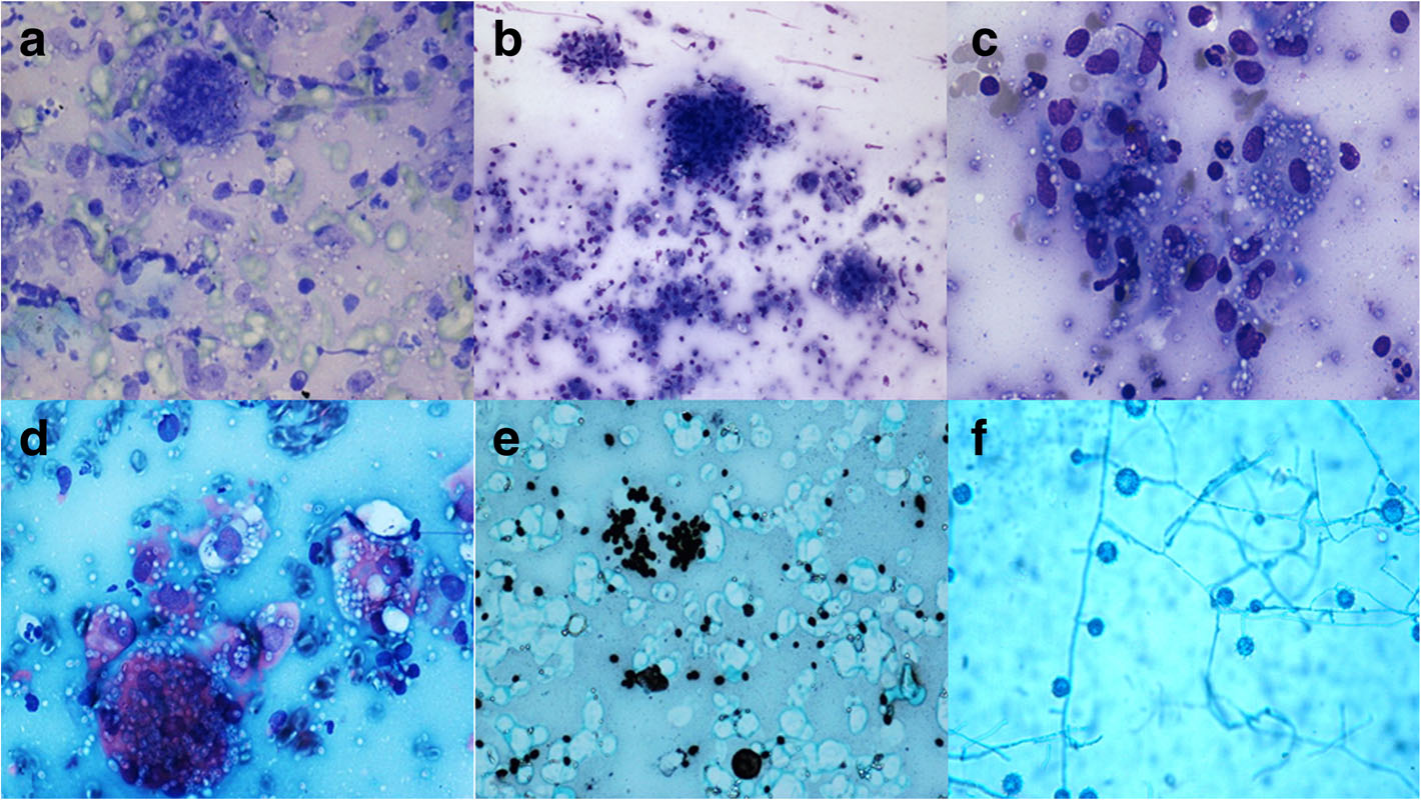
**a**, **b**, **c**, **d** Smears revealed numerous multinucleate giant cells, epithelioid cell granulomas, and mixed inflammatory cells including numerous histiocytes stuffed with ovoid yeast forms of *H. capsulatum*. (Toulidine blue, Giemsa, PAP, ×40). **e** Gomori methanamine silver showing positive staining for *H. capsulatum* yeast forms. (Gomori methanamine silver, ×40). **f** Isolated mold showing thick-walled microconidia and macrocoinida with long septate hypae (Lactophenol Blue Cotton, ×40)

Scrape cytology of the newly formed blisters over his arm was done and smears were stained with Toluidine blue (on-site), Giemsa, and Papanicoloau (PAP) stains. Smears revealed numerous multinucleate giant cells, epithelioid cell granulomas, and mixed inflammatory cells including numerous histiocytes. Background showed hemorrhage and necrosis. The histiocytes appeared stuffed with round to ovoid yeast forms. Numerous extracellular organisms were also seen (Fig. 2a, b, c, d). Ziehl Neelsen (ZN) stain for acid-fast bacilli was negative. Differentials included histoplasma, cryptococci and LD bodies (Leishmania donovani). However, the typical rod and kinetoplast of LD bodies was not seen and a definite halo was seen surrounding the organisms. A fungal stain [Gomori methenamine silver (GMS)] was positive (Fig. 2e) and mucicarmine was negative. A diagnosis of histoplasmosis was given and sample for culture was taken. Sabouraud dextrose agar (SDA) culture at 25 °C after 2 weeks, showed mold colony, which was white-brown with a cottony appearance. Lactophenol cotton blue stain of isolated mold showed thick-walled microconidia and macrocoinida with long septate hypae (Fig. 2f). Bone marrow aspiration and biopsy also revealed features of histoplasmosis. He was then  treated with amphotericin B for 2 weeks and currently under maintenance medication of itraconazole and is doing well in 6 months of follow-up.

## Discussion

Histoplasmosis or Darling disease, results from infection with *H. capsulatum var. capsulatum*. The disease has a worldwide distribution, but the largest endemic focus is in the central eastern United States, especially the Ohio, Mississippi and Missouri River valleys. Histoplasmosis is an infrequently reported disease in India and only sporadic case reports have appeared in the literature from different regions in this country with a predilection to West Bengal, which is an endemic area [4].

*H. capsulatum* grows in a mycelial form in soils, particularly those enriched by bird or bat excrement. Inhalation of sporulating mycelial fragments into lung is followed by rapid conversion to the yeast form. Histoplasmosis tends to be a widely disseminated infection involving multiple organs, particularly reticuloendothelial tissues [5].

There could be three major clinical presentations of histoplasmosis, i.e., pulmonary, progressive disseminated (PDH), and primary cutaneous. Approximately 10% of individuals infected with histoplasmosis may develop progressive disseminated histoplasmosis and 6% of patients with PDH have skin lesions. This may manifest as chronic PDH characterized by oropharyngeal ulcers with or without hepatosplenomegaly, or as acute PDH as seen in immune-compromised individuals. Acute PDH has a spectrum of clinical manifestations such as fever, malaise, cough, and weight loss and could result in the patient being treated for pulmonary tuberculosis, a more common clinical entity in our country. Disseminated histoplasmosis may also mimic leishmaniasis [6].

Widespread organ involvement is seen in 95% of cases in patients with acquired immunodeficiency syndrome (AIDS) who are infected with *H. capsulatum* and it results in protean manifestations. Elevations in lactate dehydrogenase are common. Additional frequent clinical findings include an interstitial pattern on chest X-ray, pancytopenia, and cluster of differentiation (CD)4 count <100/μL [7, 8].

The use of complement fixation serologic testing for Histoplasma antigen may be useful in identifying persons with histoplasmosis. False positives can occur with infection from other fungal agents particularly *Blastomyces dermatitidis* and *Aspergillus* [5]. Regardless of immune status, persons infected or reinfected with *H. capsulatum* will seroconvert within 4 weeks, with seroreversion within 5 years. A CD4 lymphocyte count < 300/μL also indicates an increased risk for infection. Serologic testing will demonstrate antibodies in 50–70% of cases. Antigen can be detected in urine, serum, fluid from bronchoalveolar lavage, and in cerebrospinal fluid in most cases [9].

Special stains should be used to identify the presence of *H. capsulatum* in tissue biopsies or cytologic material. Methenamine silver staining provides the best contrast and is the easiest to screen, but the yeasts may be confused with the slightly larger budding cells of candida when pseudohyphae are lacking in the latter. A periodic acid Schiff (PAS) stain helps to define the thin cell membrane or “capsule” of *H. capsulatum* and the central dot-like cell contents that form with artefactual shrinkage during fixation. Clusters of such organisms are quite characteristic of *H. capsulatum*. Microbiologic culture will provide a definitive though delayed answer [7].

The differential diagnosis includes other conditions in which parasitized macrophages are seen, especially leishmaniasis and *P. marneffei* infections [4]. The parasites in leishmaniasis lack the clear halo seen in *H. capsulatum* and have a tendency to aggregate at the periphery of the macrophage (marquee sign). LD bodies can be distinguished by a nucleus and bar-shaped kinetoplast within each amastigote and it is negative for PAS stain [4]. The *P. marneffei* yeast forms replicate by binary division and have a septate appearance, whereas *H. capsulatum* divides by budding.

Prophylaxis for *H. capsulatum* using antifungal agents has not been shown to prevent histoplasmosis. Treatment resulting in prolonged survival may include induction with amphotericin-B followed by long-term maintenance on itraconazole or fluconazole [10]. Histoplasmosis responds well to therapy, but relapses in the absence of chronic suppressive antifungal therapy. When death occurs from histoplasmosis, organ involvement is frequently so widespread that it is difficult to determine a specific organ failure as a cause of death [7].

## Conclusions

In a country like India with low socioeconomic status and overcrowding, opportunistic infections play a major role in mortality and morbidity of patients. Histoplasmosis albeit rare is one of the opportunistic infection causing organ damage and is usually missed clinically, especially in immunocompetent patients. A good fine-needle aspiration (FNA)/scrape procedure along with clinical history and special stains will help accurate diagnosis and institute early and proper treatment.

## Abbreviations

AIDS: Acquired immunodeficiency syndrome
CD: Cluster of differentiation
FNAC: Fine-needle aspiration cytology
GMS: Gomori methenamine silver
HIV: Human immunodeficiency virus
LBC: Lactophenol Blue Cotton
LD: Leishmania donovani
PAP: Papanicoloau
PAS: Periodic acid Schiff
ZN: Ziehl Neelsen
